# Case 5 / 2017 - Scimitar Syndrome and Pulmonary Sequestration in Natural Progression in a 68-Year-Old Woman

**DOI:** 10.5935/abc.20170128

**Published:** 2017-09

**Authors:** Edmar Atik, Álvaro Francisco Gudiño, Marcelo Lopes Montemor

**Affiliations:** Instituto do Coração do Hospital das Clínicas da Universidade de São Paulo, São Paulo, SP - Brazil

**Keywords:** Heart Defects, Congenital, Scimitar Syndrome, Heart Septal Defects, Atrial, Pulmonary Sequestration

## Clinical data

Tiredness and palpitations during physical work for approximately 20 years, without progression. Some episodes of small-volume hemoptysis during this period. Taking enalapril for hypertension and metformin for diabetes.

Physical examination: good general condition, eupneic, acyanotic. Weight: 56 Kg, Height: 160 cm, blood pressure (right arm): 140/90 mm Hg, HR: 95 bpm, oxygen saturation = 99%.

Precordium: apex beat was not palpable, without systolic impulses. Normophonetic heart sounds, and no heart murmurs. Liver was not palpable and lungs were clear.

### Complementary tests

**Figure 1:**
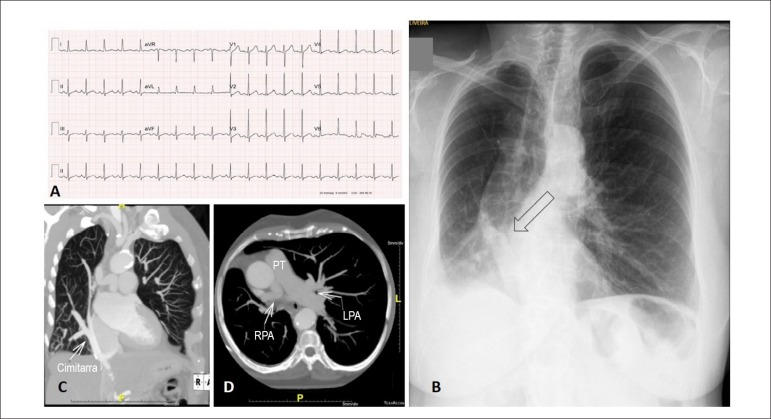
Normal electrocardiogram (A). Chest X-ray (B) In the posteroanterior view, normal-sized heart, right lung hypoplasia and scimitar-shaped vein (arrow). Computed tomography angiography shows right pulmonary vein in the shape of a scimitar (arrow) draining to inferior vena cava (C) and size contrast between the pulmonary arteries (D). PT: pulmonary trunk; RPA: right pulmonary artery; left pulmonary artery (LPA).

**Electrocardiography:** sinus rhythm, HR 113 bpm, with no signs of chamber overload, PR: 0.12, QRS: 0.89; AP = +60º, AQRS = +40º, AT = +60º (Figure 1A).

**Chest radiography:** normal-sized heart, with deviation to the right due to pulmonary hypoplasia of the right lung, which was compensated by the left lung. In the right lower lobe, there was a vascular image with the shape of a scimitar (Figure 1B).

Echocardiography: normal cardiac chambers, except for mild biatrial enlargement, normal biventricular function. Aorta = 32 mm, LA = 34, RV = 28, LV = 53, septum = posterior wall = 10 mm, LVEF = 68%. There was a very low blood flow from the LA to the RA.

**Chest computed tomography:** *situs solitus* and levocardia, and heart in dextroposition. Anomalous pulmonary venous connection at right, with drainage of pulmonary veins through a venous collector (largest diameter 11 x 10 mm), that descended to the suprahepatic segment of the inferior vena cava (scimitar) (Figure 1C).

Normal pulmonary trunk (22 mm). Dilated left pulmonary artery (27 mm) with increased pulmonary artery-to-bronchus ratio, which may indicate either redirection of the flow or increased pulmonary pressure. Right pulmonary artery was tortuous and hypoplastic, measuring 10 mm in its proximal portion and 9mm in its medial third (Figure 1D).

Enlarged branch (13 x 13 mm) from the abdominal aorta at the level of the celiac trunk with ascending course to the posterior-inferior region of the right lung ("aneurysmatic" pulmonary sequestration).

Hypoplastic right lung with compensation by the left lung.

Posterolateral diaphragmatic discontinuity, suggestive of Bochdalek hernia.

### Clinical diagnosis

Scimitar syndrome with right lung hypoplasia and pulmonary sequestration of the right inferior lobe.

### Clinical reasoning

Patient with few symptoms, without a definite clinical diagnosis, due to absence of signs suggestive of congenital heart disease and normal ECG results. Chest radiography has become paramount in the diagnosis of scimitar syndrome, which is confirmed by angiotomography. The few clinical manifestations of the disease associated with its long natural progression resulted from the small interatrial communication and low pulmonary blood flow at right.

## Differential diagnosis

Congenital heart diseases with few manifestations may have the same long term progress, including acyanogenic heart diseases with low blood flow from the left to the right, such as interatrial communication, interventricular communication, and patent ductus arteriosus (PDA).

### Management

Due to few clinical and hemodynamic manifestations of the pulmonary venous abnormality, expectant management was performed.

### Comments

Scimitar syndrome consists in an anomalous venous drainage of the right lung to the inferior vena cava associated with right lung hypoplasia, anomalies of the bronchial tree, dextrocardia, systemic arterial blood supply coming from the abdominal aorta. In one third of the cases, congenital heart diseases are concomitant, such as ventricular/atrial septal defect, PDA, coarctation of the aorta, and Tetralogy of Fallot. Other congenital disorders that may be associated with this syndrome are right diaphragmatic hernia, spinal abnormalities, hypospadias, duplicated ureter and double urethra.^1^

Scimitar syndrome was first described by Cooper and Chassinat in 1836 and its first surgical treatment was described by Kirkling et al.^1^ in 1956.

The syndrome was classified by Depuis et al.^2^ into two distinct presentations: infantile and adult. The infantile form affects children younger than one year; it usually progresses to heart failure and pulmonary hypertension, with a worse prognosis. The adult form affects both children and adults; it is usually asymptomatic and has a better prognosis.^1,2^

The name 'scimitar' (the Turkish sword) is symbolic, described by Neil et al. in 1960 due to the radiographic image showing the oriental sabre appearance of the anomalous, vertical descending pulmonary vein in the right lung.^1^

Most of the cases reported in the literature undergo surgical repair from early years to youth, and rarely in adult ages, as described in a 66-year-old patient.^3^
